# Approaches to Minimise the Neurodevelopmental Impact of Choroid Plexus Carcinoma and Its Treatment

**DOI:** 10.3390/life13091855

**Published:** 2023-09-01

**Authors:** Jenny Adamski, Vikki Langford, Jonathan L. Finlay

**Affiliations:** 1 Birmingham Women’s and Children’s Hospital NHS Foundation Trust, Birmingham B4 6NH, UK; vikkilangford@nhs.net; 2 Ohio State University College of Medicine, The Ohio State University, Columbus, OH 43210, USA; neuronc514@aol.com

**Keywords:** choroid plexus carcinoma, neuropsychological consequences, infant brain tumour

## Abstract

Choroid plexus carcinomas (CPC) are rare aggressive tumours that primarily affect very young children. Treatment for CPC typically involves a combination of surgery, chemotherapy, and radiation therapy. Whilst considered necessary for a cure, these therapies have significant neurocognitive consequences for patients, negatively impacting cognitive function including memory, attention, executive functioning, and full-scale intelligence quotients (FSIQ). These challenges significantly impact the quality of life and ultimately socioeconomic parameters such as the level of educational attainment, marital status, and socioeconomic status. This review looks at the tumour- and treatment-related causes of neurocognitive damage in CPC patients and the progress made in finding strategies to reduce these. Opportunities to mitigate the neurodevelopmental consequences of surgery, chemotherapy, and radiation therapy are explored in the context of CPC treatment. Evaluation of the pathological and biological mechanisms of injury has identified innovative approaches to neurocognitive protection and neurorehabilitation, which aim to limit the neurocognitive damage. This review aims to highlight multiple approaches physicians can use when treating young children with CPC, to focus on neurocognitive outcomes as a measure of success.

## 1. Introduction

**Figure 1:**
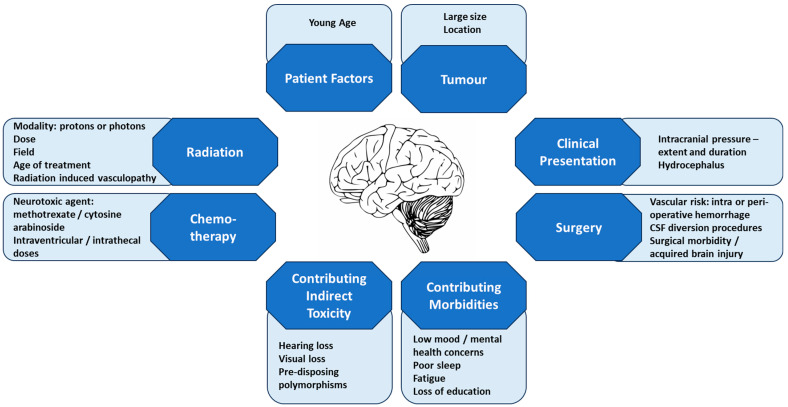
Factors Contributing to Neurocognitive Damage.

**Figure 2:**
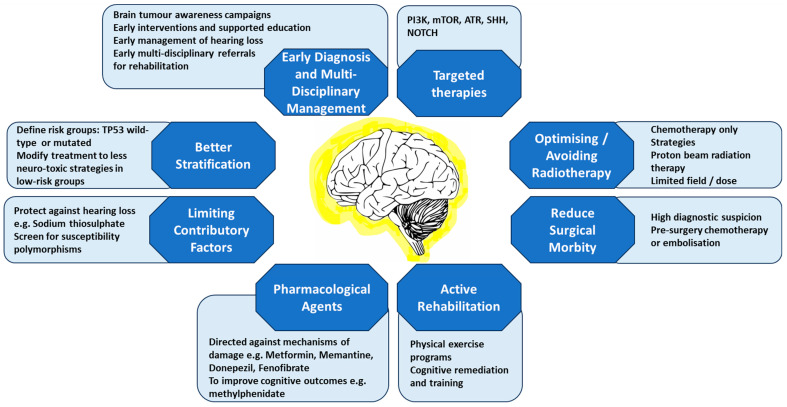
Possible Management Opportunities to Minimize Neurocognitive Damage.

Choroid Plexus Carcinomas (CPC) are rare epithelial brain tumours derived from the choroid plexus. Although reported at all ages, the vast majority occur in childhood with the median age of diagnosis being 1–2 years [1,2,3,4,5]. In childhood these are generally large, vascular, and supratentorial tumours. This profound insult at such a young age can have severe neurodevelopmental implications. Compounded by the need for aggressive treatment measures, the effect on cognition can be marked. With improving survival rates, a new focus on protecting and preserving brain function is needed. Much can be done to minimise the damage and improve the function, by considering treatment options, provision of neurorehabilitation, and the use of innovative protective measures for the developing brain. This review aims to explore the causes of neurodevelopmental insults in children with choroid plexus carcinomas (Figure 1) and considers the implications for treatment and management to minimise the adverse effects (Figure 2).

The first challenge when considering CPCs is their rarity. Occurring in approximately 0.3 per million children, all published series contain only a small number of patients [5,6]. This means that there is a lack of data on all aspects of management and the subsequent impact on outcomes. Clinical trials are challenging in such a rare cancer, with only one randomised trial performed, the SIOP CPT 2000 [7]; a second closed early due to lack of accrual (SIOP CPT 2009 [8]). It is difficult to draw significant conclusions with such small patient numbers. Comparisons are made across series, which contain different patient populations and are easily confounded. Particularly for this review, there are very few data on the long-term neurocognitive outcomes associated with CPC. Inferences are drawn from the data on other paediatric brain tumours, especially those brain tumours occurring in infancy. Unsurprisingly then, many of the strategies and approaches demonstrated here to address negative neurocognitive consequences are valid for and applicable to the treatment of other brain tumours in infants.

The second challenge is the young age at which these children present. Making up 12–20% of brain tumour patients in the first year of life, this is an especially vulnerable time of brain development [4,5]. Treatment options can cause greater adverse effects when given to younger children, particularly the use of radiation therapy [9,10,11]. Although the younger brain is often thought to have more plasticity and therefore propensity to recover from damage, the data from acquired brain injury and rehabilitation show this may not be the case, and the damage often comes at a cost to other functions. Series of brain tumour patients show that patients of younger age at the time of diagnosis are more profoundly affected [10,12,13]. Neuropsychological testing in very young children is also practically challenging, with testing methods and outcome measures inconsistent across studies, making comparisons difficult [14]. 

The third challenge is the historically poor outcome of CPC patients, with the 5-year overall survival (OS) reported to be between 21% and 74% [5,7,15,16,17,18]. Physicians have fought for a ‘cure’ at the expense of the ‘cost’ of that cure. Although there is no standard of care, treatment includes maximal safe surgery, multiagent chemotherapy, and either high-dose marrow-ablative chemotherapy with hematopoietic stem cell rescue (HDC) or radiation therapy. Chemotherapy improves survival, and chemotherapy responses are well documented with cyclophosphamide, etoposide, carboplatin, and vincristine of most value [2,3,19]. More recently, intravenously administered high-dose methotrexate-containing regimens have been added [17,18]. Due to the relative sensitivity of the TP53 mutant cells to methotrexate, this drug is being considered in future CPC protocols trying to improve outcomes in high-risk CPC patients (see Table 1) [20]. Intraventricular chemotherapy has also been used, although more in the context of relapsed disease; however, the evidence for its benefit comes from single case reports [21]. Radiation therapy is standard practice in children old enough to receive it with minimized late effects. However, the data on its benefit are conflicting [2,4,19,22]. HDC has been used as an irradiation-sparing treatment and has been shown to cure patients both in the newly diagnosed and relapsed settings [17,23]. What is not known is what elements of this treatment are essential and whether all aspects are required in all patients. Recently, treatment strategies are aiming to better stratify patients and identify lower risk patients in whom treatment can be de-escalated, potentially limiting the neurocognitive damage. In CPC patients, this stratification may hinge on disruption of the TP53 pathway. Inherent to CPC’s biology, TP53 pathway disruption is required for CPC tumorigenesis, and TP53 somatic or germline mutations are found in 50–54% patients, with a significant proportion of the remainder harbouring other alterations affecting TP53 function [18,24,25]. Prognosis is heavily dependent on TP53 status and those with a TP53 mutation, somatic, or germline, have a poor prognosis compared to those without (22–29% 5-year OS, compared with 100% 5-year OS, respectively [18,25]). Thus, there is a proportion of patients (46–50%) who has neither germline nor somatic P53 mutations (P53 wildtype) whose outcome is excellent with current treatment and for whom we should be trying to reduce late effects. 

Although the impact of having a brain tumour and its subsequent treatment on cognitive development in infants is well documented, only two studies have reported cognitive outcomes in choroid plexus tumours [15,18]. Infants with brain tumours go on to have lower intelligence quotients (IQ), slower processing speeds, and poorer memory than expected population means when compared to brain tumour patients diagnosed at an older age [9,10,11]. The cause of this is thought to be multifactorial: a consequence of young age, being in a vulnerable time of rapid time-critical brain development as well as multiple and other contributory factors including tumour location, aberrant cerebrospinal fluid (CSF) production and flow, surgery and its complications, toxic treatment (chemotherapy and irradiation), and missed developmental stimulation. What limited data there are in relation to CPC patients describe neurocognitive consequences, even when radiation therapy is not given. Lafay-Cousin et al. found that six out of eight CPC survivors had significant neurocognitive impairment, none of whom were irradiated [15]. Liu et al. found that of nine evaluable CPC patients, 57% had an IQ of more than 1 standard deviation (SD) below the normative mean, which was significantly below age expectations [18]. Fifty percent displayed impairment of behaviour and adaptative functions in at least one domain on parent-reported assessments, with attention being most commonly affected [18].

## 2. Causes of Neurocognitive Damage

The first insult to the brain is from the tumour itself and the consequences of this (Figure 1). CPCs are generally large tumours, with mean diameters of 6 cm, and tumour size is a known risk factor relating to poor cognitive outcomes [1,11]. Since these tumours arise from the source of CSF production, the choroid plexi within the ventricular system, more than 70% of patients present with hydrocephalus [28]. Hydrocephalus is caused by both obstruction of the CSF flow by the tumour and by overproduction of CSF by tumour cells (non-communicating hydrocephalus) [29]. Even if the tumour is completely removed, CSF production can continue to be abnormal, and there is a high incidence of CSF diversion procedures (up to 71%) [28]. Patients with hydrocephalus and those requiring placement of a ventriculo–peritoneal shunt have been found to have worse cognitive outcomes, independent of radiation therapy and chemotherapy [30,31,32]. It is not thought that the placement itself causes the cognitive insult; rather, it is the severity of the hydrocephalus requiring treatment, shown by improved cognitive function in patients with hydrocephalus following treatment [33]. Depending on the length of symptom duration, which can range from 0 to 269 months, this chronic pressure on the brain has already caused damage before the child reaches medical attention [16]. Although most CPCs likely arise de novo, a proportion result from malignant evolution of a benign choroid plexus lesion [34], further lengthening this period of chronically raised intracranial pressure. The usual site of CPCs in children also influences the neurocognitive impact. The most common location in the supratentorium, and therefore the subsequent focus of further treatment-related damage, results in significant sequalae [10,35]. This location of brain damage also more commonly results in seizures at presentation or following surgery, itself a risk factor for intellectual deficits [30,36]. 

Choroid plexus tumours have a substantial risk of surgical morbidity. Very vascular in nature, haemorrhage and blood loss are common at presentation. Loss of 130–182% of the child’s total blood volume has been reported, with younger patients most at risk [37,38]. There is a surgical mortality rate of up to 13%, and deaths from intra-operative or post-operative haemorrhage occur in most series [15,37,38]. IQ and adaptive functioning in infants with brain tumours assessed immediately post-surgery were significantly worse than normative expectations, thought to be a consequence of both the tumour and the surgery to remove it [35]. Peri-operative complications (such as neurologic deficits, shunt infection, subdural fluid collections, and repeat intracranial procedures) significantly increase the risk of a lower IQ and complications and are higher in those with younger age, such as CPC patients [39].

It is undisputed that therapy causes neurocognitive damage, including reduced IQ and poor performance in assessments of processing speed, attention, executive function, and memory. These neurocognitive impacts in irradiated children become more pronounced with time, with children accumulating a loss of 1 to 4 full-scale intelligence quotient (FSIQ) points per year [40,41,42,43]. The reduction in IQ is thought to be a result of a failure to learn new skills and information at the same rate as peers, and critically, this effect continues to fall with time from treatment, without reaching a plateau [41,44]. This effect is more profound the younger the patient at treatment and becomes more significant with the time elapsed from the end of treatment. All patients receiving cranial irradiation have neurocognitive consequences; however, the magnitude of the effect is dependent on the dose and size of the irradiation fields [33]. Reduced-dose craniospinal irradiation (CSI) of 24 Gy has less of an adverse impact than 36 Gy CSI, and likewise, focal irradiation is associated with better outcomes than whole brain irradiation [9,45]. As well as direct irradiation damage, survivors of irradiated brain tumours also have a high incidence of irradiation-induced vasculopathy and Moya Moya syndrome, known to additionally impact adversely IQ and executive functioning, as well as increasing the risk of strokes and thus of physical deficits [46,47].

The direct effect of chemotherapy on cognition is not as well-recognised. However, data on patients with acute lymphoblastic leukaemia treated with chemotherapy alone show adverse neuropsychological outcomes compared to controls [48,49]. Although IQ was not consistently reduced, specific deficits in academic achievement and cognitive skills including processing speed, attention, visual–spatial skills, fine motor skills, and nonverbal memory were seen [48,49]. Specific chemotherapy agents have known neurotoxicity. Methotrexate and cytosine arabinoside (araC) are toxic to the central nervous system (CNS), with both acute toxicity and worsened subsequent neurocognitive performance having been well-documented, correlating with leuko-encephalopathic changes on magnetic resonance imaging (MRI) scanning [50]. Children treated with high-dose intravenous methotrexate show significantly worse performance in neurocognitive testing, especially on the subscales of processing speed, memory, and sustained attention [51]. Children receiving intrathecal methotrexate performed worse in testing of intelligence, executive function, attention, visual perception, and short-term memory in comparison with those who did not have intrathecal treatment [52]. It is not clear which administration route carries the most risk. Likewise, araC has clear acute CNS toxicities including seizures, cerebral dysfunction, myelopathy, leuko-encephalopathy, and myelopathy following intrathecal administration [53]. Although both the SIOP CPT 2000 and SIOP CPT 2009 trials contained intrathecal araC as a treatment choice in young CPC patients with metastatic disease, the benefit of this agent has never been shown in CPC, either in vitro or in patients. High-dose myeloablative chemotherapy (HDC) with stem cell rescue did not show additional statistically significant neuropsychological consequences to those deficits already accumulated by diagnosis, surgery, and conventional-dose chemotherapy. Performance on cognitive testing prior to HDC and post-surgery and induction chemotherapy was equivalent to testing at three years following treatment and indeed 5–10 years after treatment [35,54,55]. This stability of the neurocognitive outcomes with time is in stark contrast to that seen with radiation therapy, where there is a progressive decline in IQ over time [56]. Children treated with HDC had full scale IQ, overall adaptive functions, working memory, processing speed, and verbal and nonverbal memory within average to low average ranges at a mean of 5.12 years post-diagnosis, albeit as a whole, performing below the age-related expectations [56]. Children treated with high-dose myeloablative chemotherapy and surgery do have diffusion tensor imaging changes, indicating microstructural brain tissue damage, in areas related to memory and executive functioning [57]. Despite this, the majority scored within the average or above average range on testing of memory and executive functioning, suggesting they are less affected by the injury or have more capacity for neuroplasticity [57]. Significant differences in areas of microstructural injury were seen in children who received chemotherapy, compared to those who had surgery only, suggesting a direct effect of chemotherapy [58]. Chemotherapy improves survival in CPC and is a means to reduce irradiation exposure [2]. When focusing on this comparison then, radiation-therapy-sparing protocols show significantly improved neurocognitive outcomes compared to those irradiated [30]. Although individual potential cannot be accounted for, when treated with chemotherapy alone, intelligence, academic achievement, receptive language, and visual–motor integration have a tendency to fall within the normal range, measuring average to low-average scores, with social–emotional domains also better preserved [55]. 

It is also important to recognise that cognition—defined as ‘the mental action or process of acquiring knowledge and understanding through thought, experience, and the senses[59]’—does not occur in isolation, and many other factors associated with acquired brain injury (such as a brain tumour) can affect it, either directly or indirectly, measurable or not measurable. For example, platinum agents cause permanent sensorineural hearing loss, which has been associated with reduced full-scale IQ, verbal comprehension, and working memory in cancer survivors [60]. Acquired brain injury, including that caused by a tumour and its treatment, is known to be associated with fatigue, which is often prolonged and debilitating and can have a marked effect on cognition. Sleep is often also negatively affected in brain tumour survivors, again severely affecting a person’s ability to perform functional cognitive tasks [61]. Mood and mental health should also be considered, both in the short term and often years after cancer treatment, with brain tumour survivors exhibiting higher rates of depression than sibling controls [62]. The burden of brain tumour treatment in childhood inevitably means some loss of education, more so than other cancer survivors, which often extends to years following treatment. This can lead to an overall lower educational attainment, which in turn is a key predictor of future employment, income, and social integration [63]. 

## 3. Consequences of Neurocognitive Damage

The pathological and biological mechanisms of the damage incurred by a childhood brain tumour and its treatment are beginning to be characterised, following both irradiation and chemotherapy [64,65]. Radiation therapy causes direct apoptosis of neurons, oligodendrocytes, and endothelial cells, but key to the continued cognitive deterioration is felt to be the failure of neurogenesis [66]. Neurogenesis occurs mainly in the subgranular zone of the hippocampus and the subventricular zone (SVZ) of the lateral ventricles. Here, multipotent precursor cells generate neural progenitors, which differentiate into neurons or glial cells. Radiation therapy causes a reduction in these neural stem cells, particularly in the hippocampus [66]. Of interest, before the age of 18 months, the SVZ is the site of a corridor of immature neurons migrating to the pre-frontal cortex. Damage to this area at this formative time of postnatal neurogenesis, such as can occur during surgery for choroid plexus tumours in the lateral ventricles, may have functional implications on learning and memory [67]. Oxidative stress causes microglial activation which leads to alteration of the microenvironment and an inflammatory response, with high levels of cytokines such as interleukin 6, interleukin 1β, and tumour necrosis factor [68]. This chronic inflammatory response leads to the loss of neural stem cells and the modification of neurogenesis. A loss of hippocampal volume is seen, associated with a decline in hippocampal-related functions, particularly learning, memory, and processing of spatial information. The same mechanisms of damage are described in chemotherapy-induced neurotoxicity. Studies have shown direct cell damage, oxidative stress, microglial activation, and loss of neurogenesis [69]. Chemotherapy has a particular effect on oligodendrocyte precursor cells (OPC), with a loss of oligodendrocytes and failure of OPC differentiation [70]. Consequentially, there is a failure of proper myelination and loss of white matter tracts [70]. White matter is also lost following irradiation, and MRI studies have demonstrated reduced white matter compared to age-matched controls and a relative increase in CSF [71]. Whether this is a loss of white matter or a failure to gain white matter at the appropriate rate is not known, but the reduction is correlated with both the dose of irradiation and the reduction in IQ. Radiation therapy causes disruption of vasculature, ‘leakiness’ of the blood–brain barrier, and long-term vasculopathy. This small blood vessel disease leads to ischaemia, similar to vascular dementia, which leads to an increase in glutamate and excessive N-methy-D-aspartate (NMDA) receptor excitation in the cortical and hippocampal neurons, which may lead to toxicity [72,73].

The neuropsychological outcomes of a paediatric brain tumour have been well-documented and are not detailed in this review [74,75]. The main measured result of this damage in brain tumour survivors is a lower full-scale intelligence quotient (FSIQ), documented before treatment, in those treated with surgery alone, with chemotherapy, and especially with irradiation [35,76]. Studies have highlighted a reduction in performance and verbal intelligence quotients (PIQ and VIQ, respectively), some with both PIQ and VIQ, but the PIQ is often more profoundly affected [31]. This lower IQ is now recognised to be due to a decline in core cognitive functions, primarily attention, processing speed, executive functioning, and working memory, which are key to efficient learning and academic achievement [76]. However, there are more subtleties to the cognitive challenges facing childhood brain tumour survivors, which result in lifetime functional impacts. Childhood cancer survivors are at risk of adverse socioeconomic outcomes, and consistently across studies; the worst-affected are those with childhood brain tumours, those who have received cranial irradiation, and those with a younger age at diagnosis (< 3–5 years) [77]. Compared with the general population or with sibling controls, they have lower attainment at school and are more likely to need specialist education and, overall, achieve a lower educational level [77]. Childhood brain tumour survivors are five times more likely to be unemployed than controls, with unemployment rates up to 49% [78,79]. They are less likely to obtain managerial or professional positions and more likely to have a lower income. CNS tumour survivors are ten times more likely to be receiving social services benefits than population controls [77]. 

Self-reported health-related quality of life (HRQOL) measures show overall lower scores in survivors of childhood brain tumours when compared with sibling controls [80]. Risk factors for psychological distress and poor HRQOL were lower educational attainment, unmarried status, lower annual household income, unemployment, and cranial irradiation. Childhood cancer survivors, particularly brain tumour survivors, have a significantly higher rate of hospital contacts for mental health disorders, especially emotional and behavioural disorders [81]. Parent, teacher, and self-reported measures have demonstrated difficulties in social integration, depression, anxiety, attention deficits, and antisocial behaviours [82,83]. Parents and carers report poorer social integration. Survivors have fewer friends and are less likely to form a long-term relationship or get married [31,83,84]. The poorer the neurocognitive performance, especially regarding executive function and working memory, the less likely they are to achieve these milestones [31]. In one series of brain tumour survivors, 36% were unable to live independently [31].

## 4. Minimising the Impact of Choroid Plexus Carcinoma and Its Treatment on Neurocognition

### 4.1. Optimising Current Therapy

There are opportunities to make changes to the current standard management of CPC patients, which may reduce the risk of cognitive impairment (Figure 2). 

Early diagnosis of childhood brain tumours may reduce the burden of disease, and active awareness campaigns, such as the HeadSmart Campaign in the UK, have shown reduced time to diagnosis [85]. In CPC, at presentation and before surgery, a high level of suspicion is needed to anticipate the diagnosis based on imaging. If CPC is suspected based on imaging criteria, consideration of different surgical approaches may reduce morbidity and mortality, whilst still achieving the maximal resection rate. Although not definitive, CPC can be suspected by its intraventricular site, vascularity, arterial spin labelling, and magnetic resonance spectroscopy [86,87]. Preoperative embolization in choroid plexus tumours (CPT) was found to reduce blood loss from 224% total blood volume to 96% and facilitated a higher gross total resection rate [37]. Additionally, embolization reduced the production of CSF by the tumour, although it did not change CSF diversion rates [88]. Paediatric neurosurgeons are growing less supportive of tumour embolization however, due to reports of severe acute sequelae of uncontrollable oedema and death. Alternatively, others advocate for neoadjuvant chemotherapy following limited biopsy/resection. Chemotherapy is reported to facilitate second-stage surgery by reducing tumour vascularity and blood loss [89]. In this retrospective case review, intra-operative blood loss was reduced from 96% total blood volume in those not receiving chemotherapy prior to surgery to 22% in those who did, and a higher rate of gross total resection was achieved (83% (10/12) vs. 30% (3/10)) [89]. Reducing operative risk may allow more optimal use of surgical techniques such as cortical mapping, facilitating preservation of eloquent areas where possible.

Although radiation therapy is unquestionably the most damaging treatment modality, many still consider it necessary for a cure. Certainly, there is evidence that radiation therapy is effective against CPC and that survival is better when irradiation radiation therapy is administered [2,22]. However, data are conflicting, with others showing no benefit of radiation therapy [4,19] and recent data failing to show a significant difference in outcome between those irradiated and those not, albeit in very small numbers [18]. There are patients cured without the use of radiotherapy. Again, the numbers of patients are small, and the TP53 mutation status, hence risk, is not known for many, but a percentage of patients (36–66%) do survive without irradiation (12 out of 33 patients [7], 8 out of 12 patients [15], 5 out of 12 [17]). This suggests radiation therapy is not essential for cure and therefore stratification of lower risk patients to treatment without irradiation is possible. Particularly in patients with TP53 wildtype tumours, where reported survival is excellent (80–100%) [18,25], future strategies might consider whether irradiation can be avoided, even in older patients. Tabori et al. found that 14 of 16 TP53 wildtype patients survived without radiation therapy irradiation or high-dose marrow-ablative chemotherapy, receiving only several cycles of standard-dose chemotherapy [25]. Particular consideration must be given to those with germline TP53 mutations (the Li–Fraumeni cancer predisposition syndrome) the incidence of which is high in CPC, estimated at 30–100% [25,90]. Although the presence of a TP53 mutation confers a poorer prognosis and therefore higher risk, the outcome is worse in Li–Fraumeni Syndrome (LFS) patients if radiation therapy is used (18% versus 58% OS at two years), likely a consequence of the DNA-damaging effect of irradiation in patients predisposed to cancer [8,91]. If radiotherapy is unavoidable (for example, if there is clearly residual unresectable viable tumour despite chemotherapy), it may be possible to reduce the fields of irradiation to focal fields rather than craniospinal irradiation (CSI) in localised disease, as analysis of the SIOP CPT Registry data demonstrated equivalent outcomes in localised disease (unpublished, personal communication). In the future, there may be other strategies that might be used, for instance, using agents that potentiate or act synergistically with irradiation and, thus, may allow the dose of irradiation to be reduced (e.g., ataxia telangiectasia mutated (ATM) inhibitors [92]). Although generally higher radiotherapy doses have been used for CPC patients, (36 Gy CSI with 54 Gy focal tumour boosts), doses of 18 Gy to 24 Gy have a significantly lower adverse cognitive impact [45]. Of course, the neurotoxicity of such potentiating agents is not yet known. Consideration must be made to the mode of radiation therapy, whether proton or photon beam radiation therapy. When compared with conventional photon irradiation (XRT), proton irradiation (PBRT) delivers the same target dose but overall a lower entrance dose and nearly no exit dose, reducing the area of irradiated brain [93]. A recent meta-analysis comparing XRT and PBRT showed those receiving PBRT performed significantly better across varied neurocognitive parameters including full-scale IQ, verbal comprehension, perceptual reasoning and processing speed indices, verbal working memory and working memory index, visual motor integration, verbal memory, and focused attention [94]. With the exception of working memory and processing speed, which varied across studies, patients who received PBT showed stable neurocognitive functioning with time, including IQ and executive functioning [93,95]. In those too young to receive irradiation or following irradiation-sparing strategies, high-dose marrow-ablative chemotherapy (HDC) with haematopoietic stem cell rescue has been used [17]. Salvage post-relapse using HDC and protocols for newly diagnosed children with CPC using HDC have good comparative survival rates (HS I- III Protocols, 12 patients, 64% 5-year OS) [17,23]. Although less neurotoxic than irradiation, with neurocognitive functioning scoring within the low average to average range and no drop in neuropsychological outcome measures demonstrated pre- and post-HDC, there is a higher cumulative chemotherapy burden, therefore with higher (if not measurable) indirect and direct treatment effects impacting cognition [54,56]. We do need to consider which patients may benefit from HDC and which do not need such an intensive strategy. Survival without HDC or radiotherapy is reported, and key will be defining the cohort of patients who do not need either of these strategies [7,15]. 

Metastatic disease in CPC patients is a poor prognostic factor [96]. In young patients with metastatic disease, intrathecal chemotherapy (IT) has been used as CNS-directed therapy, and there are accumulating data to suggest this to be an effective administration route in brain tumour patients [97,98]. However, there is no current evidence to show an additional benefit of IT in up-front treatment or in the recurrent setting, although some reported efficacy has been shown in the relapsed CPC [21,99]. In the SIOP CPT 2000 trail, where some metastatic patients had IT araC or IT etoposide, no survival benefit was seen (unpublished, personal communication). Given its additional toxicity, it may be that this treatment, in the absence of any formal clinical trial, should be reserved for those patients who have failed first-line therapy. 

The lack of data and inability to conduct and complete clinical trials hinder change of treatment strategies, with clinicians generally favouring ‘overtreating’ rather than ‘undertreating’ given the poor outcome in CPC patients. However, TP53 mutation is emerging as the key prognostic factor, and therefore, better stratification of patients using TP53 mutation status may allow the safe reduction in the treatment of some patients. Better characterisation and confidence in CPC molecular subgroups may help identify clearly high-risk patients and so further stratify treatment [26,100,101]. Other than the clear dysregulation of the TP53 pathway in CPC, which is not currently targetable, no driver mutations or alterations have been found to direct new or targeted agents [27]. This may largely be due to the very limited number of samples available, making collecting biological data as part of future clinical trials an imperative. As the molecular landscape of CPC becomes more defined however, and with accumulating pre-clinical data, possible novel therapeutic targets are considered, including phosphatidylinositol 3-kinase (PI3K), mammalian target of rapamycin (mTOR), ataxia telangiectasia and Rad3-related protein (ATR), platelet-derived growth factor receptor (PDGFR), sonic hedgehog (SHH), and NOTCH [102]. In an individual case, molecular profiling found altered biological pathways, which were targeted, and the patient showed a remarkable tumour response (92% reduction in tumour size) and derived a clinical benefit [103].

### 4.2. Pharmacological Agents to Protect against Cognitive Damage or Aid Cognitive Recovery

There are several agents in development which aim to ‘protect’ against the damage caused by irradiation or chemotherapy or may help mitigate the damage once it is completed. One hopeful area of development is aimed at the mechanisms of injury within the brain—toxic activation of microglia or the NMDA receptor, chronic inflammation, or the loss of neurogenesis. Metformin, an antihyperglycemic agent also found to increase neurogenesis, has been shown to promote the regeneration of neural precursor cells in mice. In a pilot crossover study in children at least two years after irradiation for a brain tumour, metformin treatment improved working memory [104]. These promising results need further exploration in larger and longitudinal studies. Memantine, an NMDA receptor antagonist, has been evaluated in prospective clinical trials in adult patients with brain metastases undergoing irradiation and resulted in a significant delay in cognitive decline, reducing the rates of decline in memory, executive function, and processing speed [72,105]. In another pilot study, patients previously treated for a paediatric brain tumour (1.9 to 11.9 years from the end of irradiation) received the acetylcholinesterase inhibitor, donepezil [106]. The cholinergic system has a role in neuronal differentiation and synapse formation and is deficient in areas of ischaemia. Although small numbers, improved executive functioning was found, warranting further investigation [106]. Microglial-mediated neuroinflammation is also an attractive target to reduce cognitive damage, and several animal studies with agents inhibiting microglial activation, such as fenofibrate and minocycline, have shown positive results justifying further exploration [107,108].

Another strategy is to protect against the toxicity of chemotherapy, by reducing the morbidities also adversely impacting cognition. Hearing loss is a significant contributor to cognitive deficits in brain tumour patients. Although not clinically available yet, it may soon be possible to screen patients for high-risk genetic polymorphisms, which determine genetic susceptibility to sequelae, and therefore focus interventions on these patients [109]. The ototoxicity of the platinum agents may be reduced with the use of oto-protectants, such as STS (sodium thiosulphate). STS has been shown to significantly reduce hearing loss (29% in those treated with STS compared with 56% without [110]) and is being tested in clinical trials for efficacy and safety [109,110,111]. Early detection and intervention of hearing loss is also vital to optimise hearing and reduce any impact, using amplification and hearing aids, which are becoming increasingly sophisticated as well as MRI compatible. 

Another strategy is to improve the functional cognitive outcomes of patients following treatment. Psychostimulants, most commonly methylphenidate, have been shown to improve attention in children previously treated for brain tumours as well as other groups of children experiencing acquired brain injury [112]. As well as improving vigilance and focus of attention, methylphenidate can improve reaction time, paired-associate learning, and perceptual efficiency [113]. Methylphenidate treatment in adult brain tumour patients significantly improved cognitive function (including psychomotor speed, memory, and executive functions) as well as mood and activities of daily living [114]. In children, improved parent and teacher behavioural scores and social skills ratings were seen [71]. Although results have been promising, at least for a subset of patients, it is not clear whether this response can be sustained and who will gain the most benefit.

### 4.3. Neurorehabilitation and Cognitive Remediation

Rehabilitation following treatment, after the brain insult, can also have a dramatic effect on cognition and particularly functional outcomes. Physical exercise, as well as improving cardiovascular health, mobility and overall fitness, can positively change the brain structure in childhood brain tumour survivors; a structured exercise program in children one to 10 years after irradiation for primary brain tumours resulted in increased thickness of the motor and sensorimotor cortices and increased volume of the underlying white matter [115]. With this exercise program, the hippocampal size increased and improved reaction time was seen [116]. Although there remains much to learn about which components and cardiovascular parameters are important, evidence is accumulating that physical exercise can affect functional cognition even years after the insult, with improvements in attention and task accuracy [117].

Cognitive remediation, the systematic retraining of the brain following acquired injury to improve cognitive function, is an established practice in many neurological conditions and in children having sustained traumatic brain injury. Programs are designed to improve functional cognitive parameters such as processing speed, attention, and working memory. Whilst these have been shown to significantly improve attention and academic achievement, these changes are often not generalisable to other areas of function or sustained over time, suggesting a need for ongoing training, which is often an unacceptable burden [118,119]. With the expansion of digital technology, cognitive training has become more accessible to children as computer-based games, apps, or interventions thatmay become more palatable. It may also be possible to identify those at most risk and target interventions to them. Specific genetic polymorphisms, such as the catechol-O-methy transferase (COMT) polymorphism, have been identified in patients who are more susceptible or resilient to neurocognitive damage [120].

## 5. Conclusions

Choroid plexus carcinomas are rare tumours occurring in a very vulnerable young population. The treatment emphasis until now has been on attempting cure at all costs; however, it is now established that a cohort of TP53 wildtype patients have an excellent prognosis. For these patients, it may be possible to reduce treatment and hence limit the neurocognitive damage that the tumour and its treatment cause. Even in high-risk CPC patients, much can be done to minimise the neurocognitive effect by carefully considering the evidence behind what we do and protecting and rehabilitating where possible. It is clear there is a paucity of data with which to inform best practice in CPC management. The rarity of this disease calls for international collaboration to be able to draw meaningful conclusions. International clinical trials using risk stratification to test the safety of treatment reduction in patients with less risk are welcome, but it is also vital that neurocognitive impact is considered as an outcome measure in those whose prognosis remains poor and who require intensified treatment. Accurately measuring the difficulties faced by these children will help us address them. Crucially, such trials must include exploratory aims, focusing on biological sample collection, molecular subgrouping, and target identification. Although neurooncologists are cognisant of cognitive damage, it is often felt unavoidable in the quest for cure. Even with less toxic treatment choices, clinicians have been resigned to a certain amount of damage being inevitable and have almost accepted this. However, there is currently an active movement fighting the impact of brain tumours and their treatments, by protecting against and/or actively rehabilitating from damage. We must learn from our colleagues in other fields, already protecting against neurodegeneration and brain damage, to bring these benefits to children with brain tumours. It is unrealistic to believe that neurocognitive damage can be avoided in CPC patients; however, changes to most points of care may lessen the impact of the tumour and its treatment on neurodevelopment. Proactive schemes to promote awareness and early diagnosis, careful consideration and optimisation of currently utilised treatment options, and active rehabilitation can be undertaken now. Other strategies, such as using agents to actively protect against neurocognitive damage and developing less toxic treatment strategies, require exploration but should be forefront of research agendas if we are to improve the quality of survival and properly weigh the “cost” of the cure for children with CPC.

## Figures and Tables

**Table 1:** Risk factors in choroid plexus carcinoma.

Low Risk Features	High Risk Features
TP53 Wildtype	TP53 Mutated (Somatic or Germline)
Paediatric DNA Methylation Subgroup A [26,27]	Paediatric DNA Methylation Subgroup B [26,27]
Age > 3–5	Age < 3–5
Localized Disease	Metastatic Disease
Gross Total Resection	Residual Disease

## Data Availability

No new data were created or analyzed in this study. Data sharing is not applicable to this article.
